# Redefining surgical workflow efficiency: evaluation of a novel laparoscopic multi-tool prototype

**DOI:** 10.1007/s00464-025-12314-y

**Published:** 2025-11-03

**Authors:** Fabian Haak, Hans-Michael Tautenhahn, Uwe Scheuermann, Daniel Seehofer, Antonello Forgione, Jacques Marescaux

**Affiliations:** 1 Division of Hepatobiliary Surgery and Visceral Transplant Surgery, Department of Visceral, Transplant, Thoracic and Vascular Surgery, University Hospital Leipzig, Leibnizstraße 11, 04105 Leipzig, Germany; 2 IRCAD (Institut de Recherche contre les Cancers de l’Appareil Digestif), Louis Pasteur University, Strasbourg, France

**Keywords:** Minimally invasive surgery, Laparoscopic instruments, Surgical workflow, Usability testing, Prototype evaluation, Ergonomics in surgery

## Abstract

**Background:**

Minimally invasive surgery improves patient outcomes but introduces ergonomic and workflow inefficiencies due to frequent instrument changes. Symphera GmbH developed a novel laparoscopic prototype enabling intracorporeal tool tip switching, aiming to enhance efficiency and reduce surgical interruptions.

**Methods:**

Seven experienced surgeons evaluated the prototype during standardized tasks using excised animal tissue in a simulated laparoscopic environment. The tasks assessed precision, ergonomics, and tool-switching functionality. Quantitative data were obtained by video analysis; qualitative insights were gathered via semi-structured interviews and a post-procedural questionnaire.

**Results:**

All participants completed the tasks successfully, with no safety concerns. A total of 148 tool changes were performed. The automated exchange sequence required a constant mechanical time of 2.6 s, with a median overall changing time of 3.4 s. A total of 148 tool changes were performed. Tool-changing errors occurred in 4.73% of the changes, and 6.08% of changes resulted in software errors that required reboots. Changing time had no significant impact on overall task duration. The precision of monopolar application was high, and all targets were accurately reached. Questionnaire responses rated tool exchange positively, while ergonomics were judged moderate; female participants highlighted weight and comfort limitations, reflecting gender-related differences in usability. Qualitative analysis revealed both device improvement needs and strong enthusiasm for its potential to streamline workflows and enhance surgical autonomy.

**Conclusion:**

The prototype was safe, functional, and well-received. While software instability and ergonomic refinements remain necessary, the system demonstrated feasibility for rapid intracorporeal tool switching and showed promise for reducing operative inefficiencies. Further technical development and clinical trials are warranted to establish its clinical and economic value.

**Supplementary Information:**

The online version contains supplementary material available at 10.1007/s00464-025-12314-y.

Minimally invasive surgery (MIS) has transformed surgical practice over recent decades by reducing postoperative morbidity, shortening hospital stays, and accelerating recovery [1–3]. However, despite these advantages, MIS is still hindered by persistent challenges such as operative inefficiencies, frequent instrument exchanges, surgeon fatigue, and rising procedural costs [4–10]. Studies have consistently shown that prolonged operative time—often exacerbated by repeated extracorporeal tool exchanges—not only increases hospital expenses but also contributes to higher complication and infection rates [11–17]. As healthcare systems push toward value-based care and surgical efficiency, there is a growing need for novel technologies that enhance workflow without compromising safety or outcomes. One such innovation is the Symphera System, a laparoscopic multi-tool designed to automate in-body instrument switching and reduce the cognitive and ergonomic burden on surgeons while optimizing cost and time efficiency. Additionally, it promotes surgical autonomy, reducing the reliance on assistant staff.

The present study evaluates an advanced prototype with surgical professionals in a structured usability setting. We aimed to test the precision and reliability of the system during simulated surgical procedures and to gather detailed user feedback on usability, workflow integration, and clinical adoption potential.

## Materials and methods

### Prototype

**Fig. 1:**
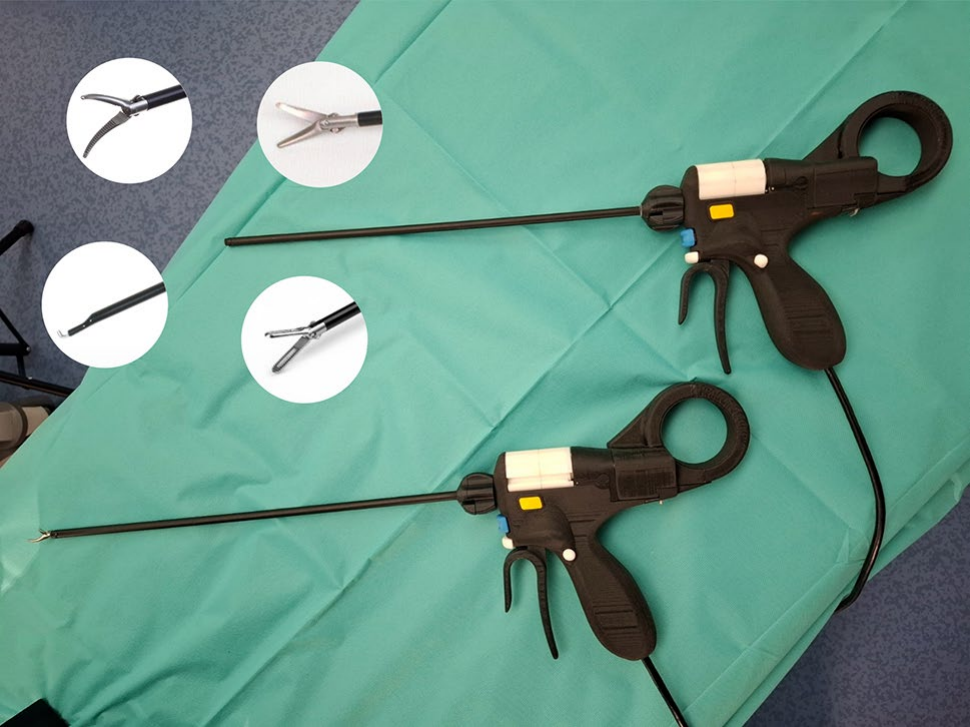
Prototype 4.2: The barrel is white and contains the different tool tips that can be introduced into the proximal end of the device once chosen

The tested prototype represents the fourth generation of its kind (Internal Development Number 4.2). (Fig. 1) This hand-held laparoscopic instrument fits through a 8-mm trocar and weighs approximately 300 g. The device measures 54 cm in total length with a maximum height of 19 cm, while the shaft—which is introduced through the trocar—has a length of 31 cm (excluding any extended tool tip). The instrument enables the user to change tool tips intracorporeally—without removing the device from the body or altering its position. Tip exchange is fully automated: once a button on the handle is pressed, the mechanism retracts the current tool tip, rotates the internal barrel to the desired position, and reintroduces the selected tip to the instrument’s proximal end. The automated exchange sequence itself requires a constant time of approx. 2.6 s, representing the raw mechanical change. Additional time may occur depending on how long the user takes to toggle through the barrel positions before confirming the desired tip. All essential functions are controlled directly on the device, allowing operation entirely within the sterile field and without altering the surgeon’s grip.

The device’s integrated software coordinates the motorized mechanics, ensuring smooth tool transitions. In addition, it communicates with a visual interface displayed on an external screen. This screen provides real-time orientation by indicating the barrel position and the corresponding tool tip currently selected. The barrel accommodates up to eight tool tip positions, which can be preconfigured by the user according to procedural needs.

### Participant details

We recruited seven surgeons (1 general director and six consultants) at IRCAD France. The group consisted of 5 male surgeons and two female surgeons. The duration of their surgical experience ranged from 6 to 30 years. The median experience was 10 years, with an IQR of 7.5 years. The median age of the participants was 37 years, with an IQR of 13 years. Six of the seven surgeons’ specialties were general surgery, and one gynecologist completed the group.

This study followed a formative, pre-clinical usability evaluation approach. The sample size of seven participants aligns with established usability research principles, particularly Nielsen’s heuristic, which suggests that testing with three to five users is sufficient to identify the majority of usability issues in formative testing. Including seven participants ensured a broad coverage of the potential problems while accounting for inter-individual variability, particularly important in a surgical context with varied levels of experience [18].

### Experimental setup and further instruments

**Fig. 2:**
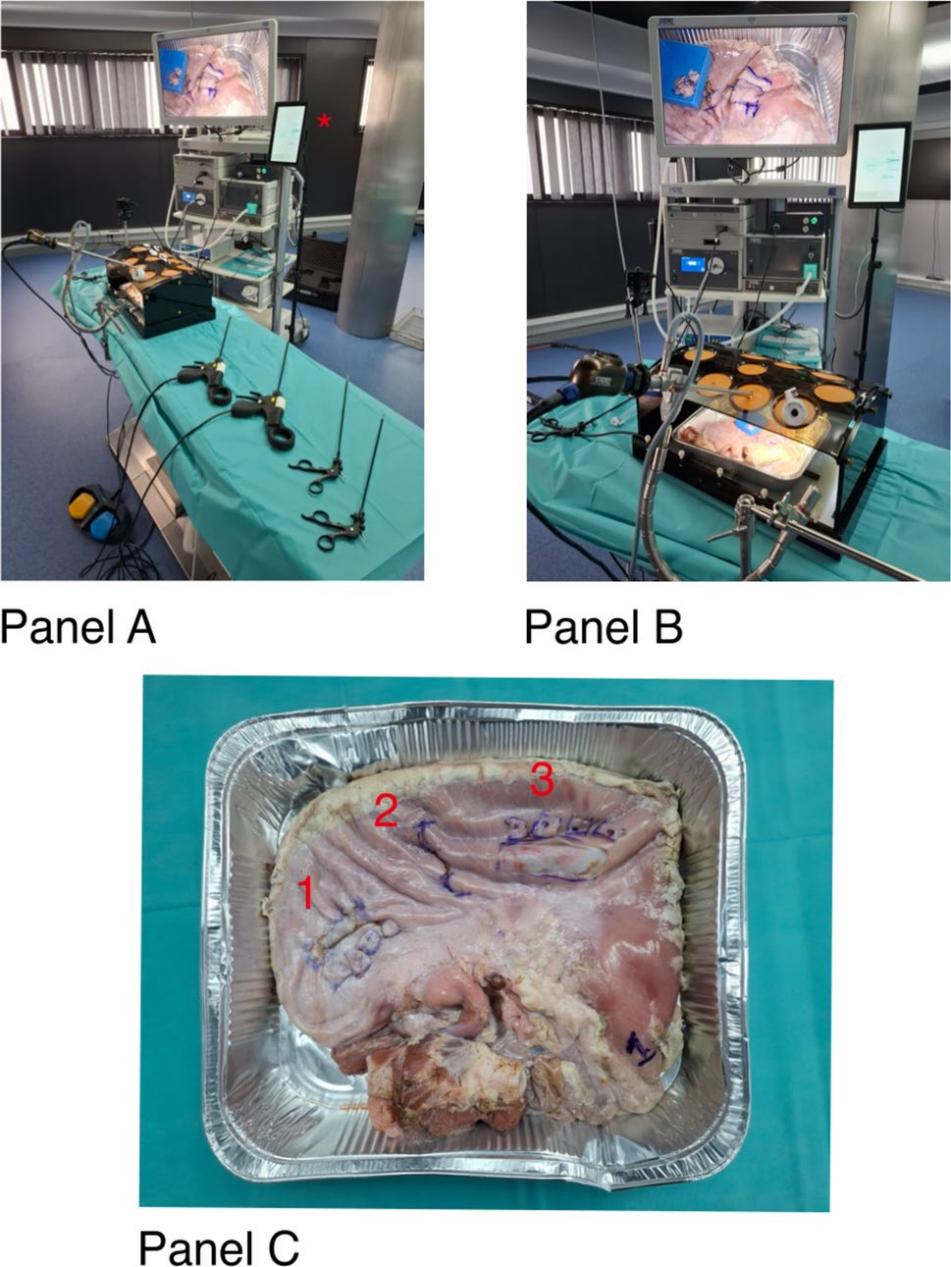
Panel **A**: Setup view 1, Additional screen for tool selection is marked with an asterisk; Panel **B**: Setup view 2; Panel **C**: Task overview on porcine tissue, 1 = Task 1, 2 = Task 2, 3 = Task 3

A surgical training station at IRCAD Strasbourg was used, consisting of a torso model with various trocar openings, a standard laparoscopic camera system (KARL STORZ SE and Co. KG, Germany), and a monitor. In addition to this laparoscopic camera, two external cameras were employed to record the participants and operator behavior. A conventional forceps (KARL STORZ SE and Co. KG, Germany) was used as an extra instrument. Participants tested the laparoscopic tool prototype by performing standardized tasks on excised animal stomach tissue (Fig. 2).

### Task explanation

Three tasks were designed to evaluate the prototype’s functionality, ergonomics, and precision.

#### Task 1: square tissue excision and transfer

Participants were required to cut out four premarked squares of tissue. Each square had two sides pre-cut, requiring the surgeon to complete the excision with two additional cuts for the first square and only one additional cut for each of the remaining three. After excising a square, the participant switched to the laparoscopic grasper tool to transfer it into a designated target box. This cycle was repeated until all four squares were successfully excised and deposited.

#### Task 2: precision cutting with monopolar energy

Participants were instructed to cut along a pre-marked line, which was divided into three zones:

- Zone 1 and Zone 3 were to be cut using laparoscopic scissors.
- Zone 2 was to be cut using the monopolar hook with electrocautery.

#### Task 3: targeting, cutting, and transfer

This task expanded on Task 1 by adding an additional precision step. Before excising each square, participants had to use the monopolar hook to target and activate energy in a premarked circular area within the square. After completing this step, they cut out the square along the pre-marked lines using scissors and transferred it to the target box with the grasper.

A picture of the task setup and results can be viewed in additional references 01.

Data collection methods:

Two primary data collection methods were employed: video analysis and semi-structured interviews.

#### Video recording and task analysis

All participant tasks were recorded using three different high-definition video cameras:

- Operating field: KARL STORZ SE and Co. KG, Germany, high-definition camera for laparoscopic surgery, focusing on the operating field and adjusted by each participant separately depending on their preference
- Experimental Setup: Samsung Galaxy S20 FE camera with 2400 × 1080 pixels on a camera stand, focusing on the whole experimental set-up
- Symphera prototype and hand movements: AKASO EK7000 Pro Action Cam with 4 K resolution, 30FPS, and 20MP, focusing on the hand movements of the participants

The recordings were analyzed to measure

- Task completion time for each participant and changing times when instruments were switched
- Errors such as tissue damage or deviations from the premarked lines.
- Device malfunctions, including mechanical failures or reboot necessity of the prototype software

Two evaluators independently reviewed video footage to ensure accuracy and consistency in the assessment. The mean was calculated between the measured times of evaluators one and two and used for further analysis. In addition, independent authors with no financial or institutional conflict of interest verified the accuracy of the measurements to ensure unbiased and reliable data evaluation.

### Qualitative interviews

Semi-structured interviews were conducted and recorded with the participants. Transcriptions were generated using Otter.ai, an automatic speech recognition (ASR) tool. Subsequent qualitative analysis was performed using MAXQDA (VERBI Software, 2024), a qualitative data analysis software [19]. An inductive coding approach was applied. MAXQDA facilitated the systematic organization, retrieval, and thematic analysis of data to interpret patterns in participant feedback.

### Quantitative survey

A post-procedural questionnaire was performed. A five-point Likert scale and a comment section were used to assess 15 items.

### Statistical analysis approach

All statistical analyses were conducted using R (version 4.4.2, R Foundation for Statistical Computing, Vienna, Austria). Descriptive statistics were used to summarize task completion times, instrument-changing times, and error rates. Due to the non-parametric nature of the data, medians and interquartile ranges (IQR) were reported for continuous variables.

Spearman’s rank correlation coefficient was calculated for each participant and task to explore trends in instrument-changing times across repeated trials within each task. A significance level of *p* < 0.05 was used.

To assess the relationship between instrument-changing time and total task duration, we applied generalized additive models (GAM) and generalized additive mixed models (GAMM) using the mgcv package. GAMs were used to model potential non-linear associations, and GAMMs accounted for participant-level random effects. Model fit and significance were assessed using *F*-tests, *p* values, and adjusted *R*^2^ where applicable.

## Results

All seven participants completed all three tasks. There were no safety issues concerning the prototype. The median time required for task one was 241 s or 4.02 min (IQR 154); for task two, 196 s or 3.27 min (IQR 79.8); and for task three, 296 s or 4.93 min (IQR 93).

All participants performed a total of 148 tool changes across all tasks. Seven changes (4.73%) were made to the wrong tool, which had to be corrected by a second change. Nine software errors occurred during the 148 changes (after 6.08% of the changes), requiring a reboot of the prototype. Rebooting required approximately 7 s, with 5 s needed for the console’s reboot sequence and around 2 s for pressing the console’s buttons to initiate the reboot sequence.

**Fig. 3:**
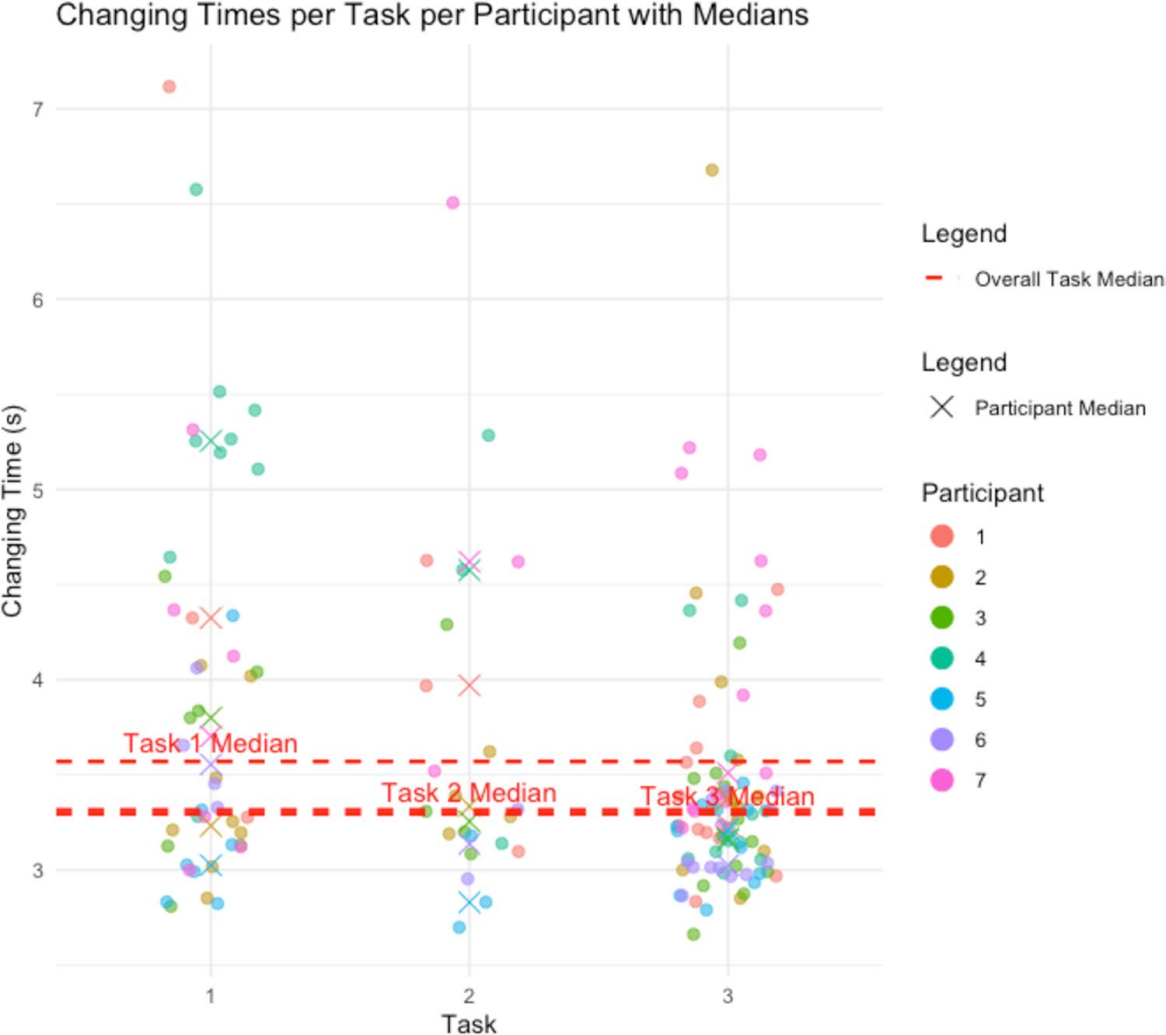
Duration for each instrument change per participant and task

Figure 3 shows the duration for each instrument change per participant and task. The median changing time for Task 1 was 3.57 s (IQR 1.28), for Task 2, it was 3.32 (IQR 1.06), and for Task 3, it was 3.30 (IQR 0.43).

**Fig. 4:**
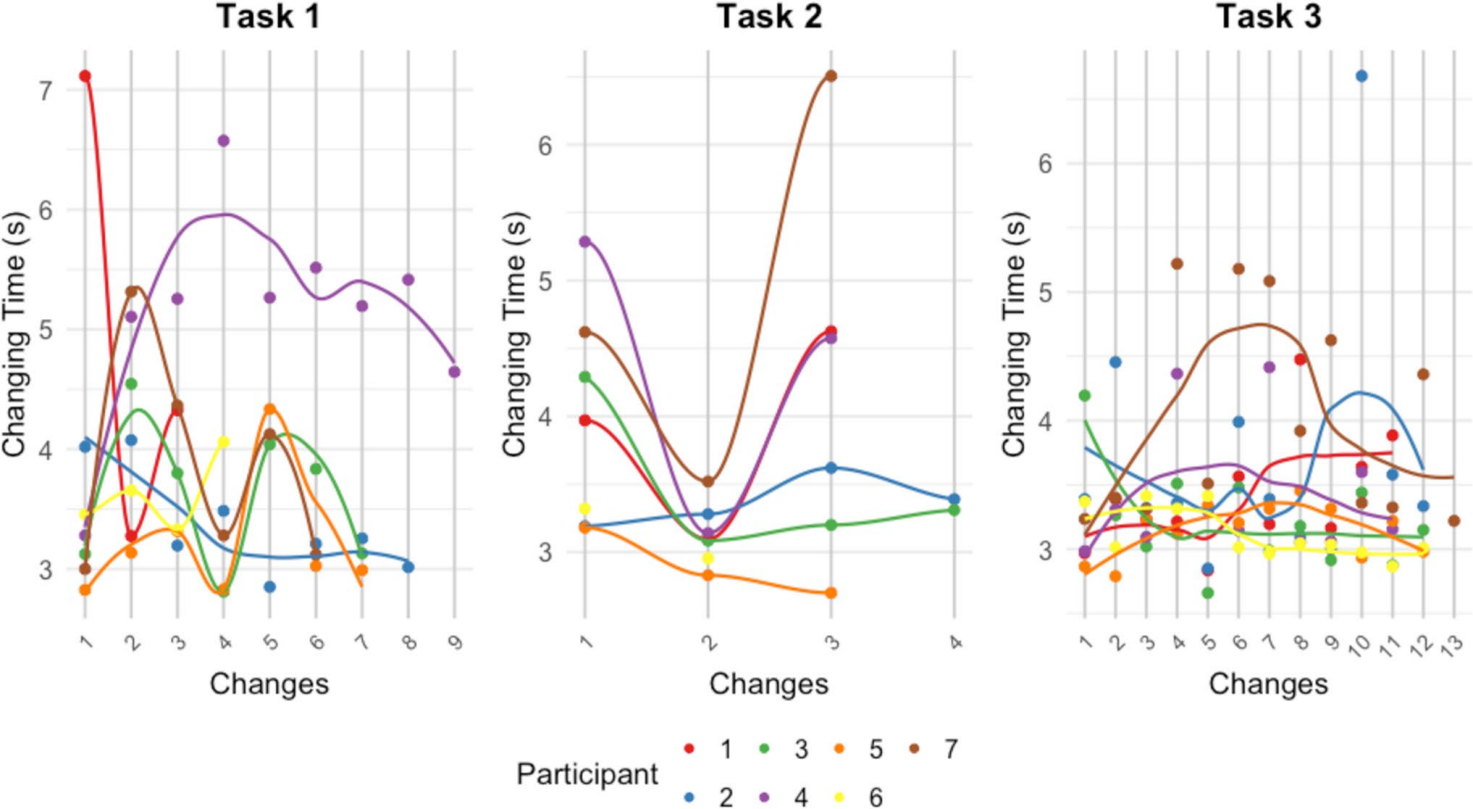
Changing times throughout the task per participant

Figure 4 shows the changing times throughout the task per participant. We tested for trend per participant and task using Spearman correlation. All Spearman correlations were nonsignificant except for participant 6 in task 3. Here, we measured a significant negative Spearman correlation of − 0.651 (*p* = 0.02).

**Fig. 5:**
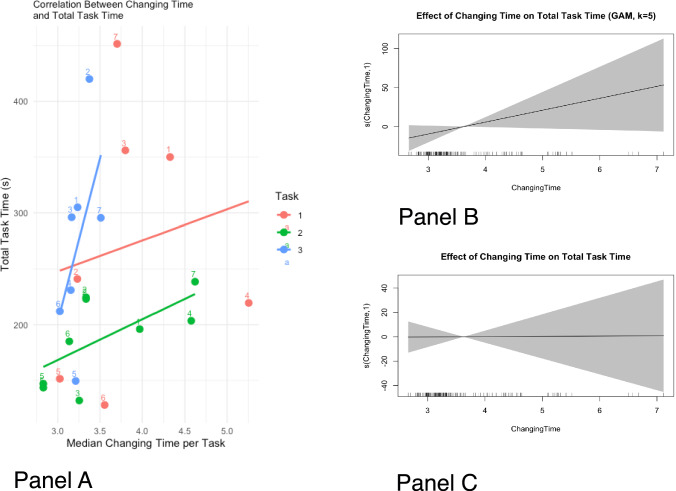
Panel **A**: Association between median changing time per participant and total task time; Panel **B**: Results of GAM model; Panel **C**: Results of GAMM model

Figure 5, Panel A, shows the association between median changing time per participant and total task time. To evaluate this relationship, we applied GAM—a statistical method that allows flexible, non-linear modeling of relationships between variables—and GAMM, which extends GAMs by incorporating random effects to account for individual-level variability, such as differences between participants. The GAM model (Fig. 5, Panel B) suggested a near-linear relationship, but the effect was not statistically significant (*F* = 3.195, *p* = 0.0754, adjusted *R*^2^ = 0.0148). The GAMM model was fitted with participant-level random effects to account for individual differences. The GAMM confirmed that there was no significant impact of Changing Time on Total Task Time (*F* = 0.157, *p* = 0.698, edf ≈ 1.004, Fig. 5, Panel C), suggesting that the weak trend in the GAM was likely due to inter-individual variability.

**Fig. 6:**
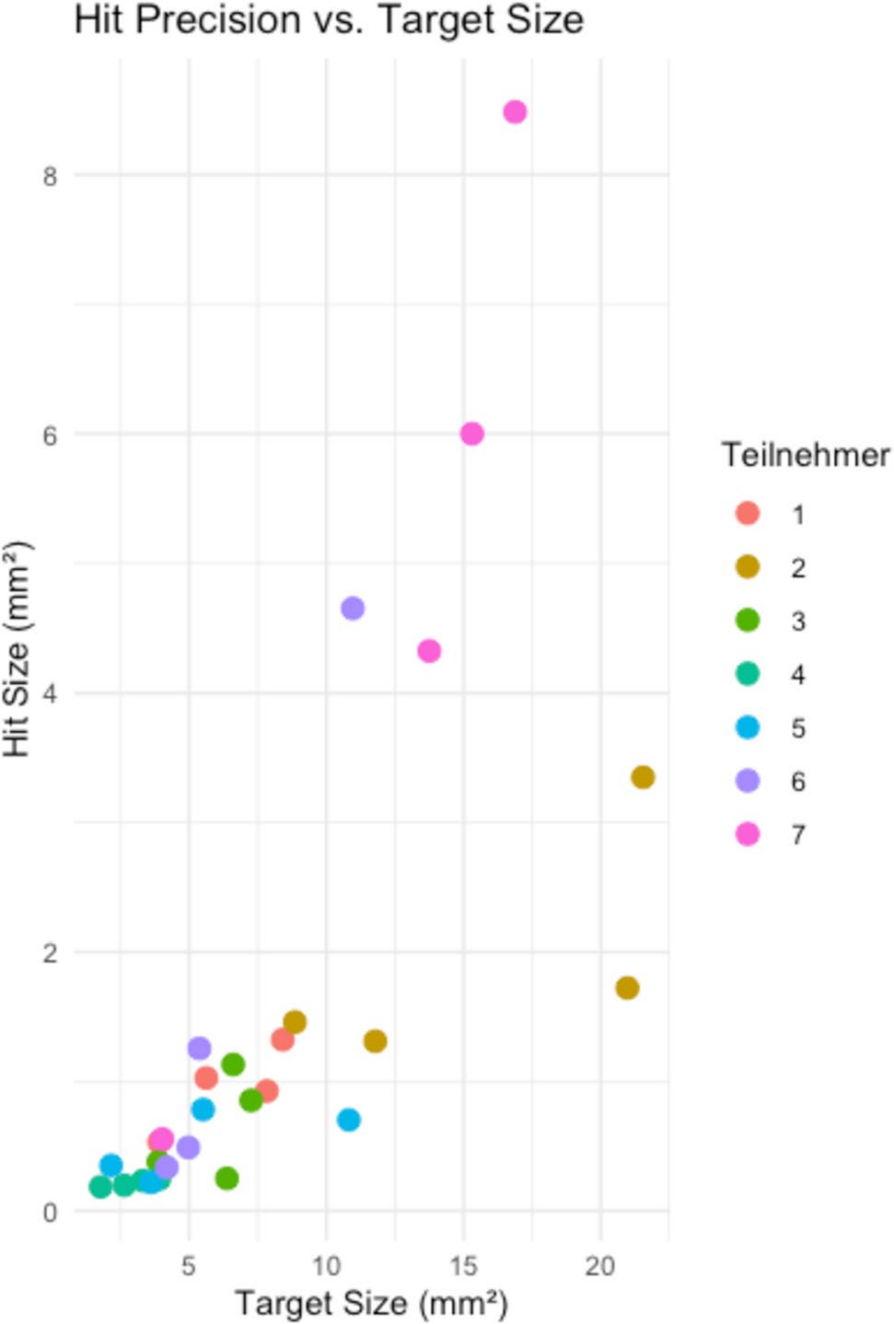
Results of monopolar precision test

Figure 6 illustrates the results of the monopolar precision test. All monopolar applications were successfully placed on target, and no safety issues were observed. The graph illustrates the size of the applied monopolar energy on the tissue in relation to the specific target size. The median applied energy area was 0.82 mm^2^ with an interquartile range (IQR) of 1.01 mm^2^.

The post-procedural questionnaire queried the following categories: general concept, operational concept, and workflow. All seven participants completed the questionnaire. The most prominent outcomes concerning the expected benefits of the device are shown in Table 1.

**Table 1:** Results of the post-procedural questionnaire

Question	Average	STDW	Likert scale
How much do you think the focus on the operation improves because you look away from the screen less?	1.5	0.55	1 (a lot)—5 (very little)
How did you perceive the instrument changeover times?	3.9	0.90	1 (very slow)—5 (fast)
How much does the device improve your overall workflow during your daily surgeries?	1.7	0.76	1 (a lot)—5 (very little)

The complete questionnaire, including all answers, is provided in Additional Reference 02. All answers were reported on a standard Likert scale (1–5) with response rotation, used to reduce habituation and response bias by varying the direction of the Likert scales. In summary, ergonomics were rated moderate (weight 2.6 (Answer 2: rather light–Answer 3: exactly right), hand feel 2.4 (Answer 2: good–Answer 3: neutral), and long-term use 2.1 (Answer 2: good–Answer 3: neutral)), while the tool exchange interface (4.0 (Answer 4: good–Answer 5: very good)) and speed of changeover (3.9 (Answer 3: neutral–Answer 4: rather fast)) were perceived positively. Importantly, the overall workflow impact was rated favorably (1.7, where lower values indicate greater improvement (Answer 1: a lot–Answer 2: a little bit)). Key areas for refinement included ergonomics, comfort, and screen integration. Notably, gender-related differences were observed: female participants rated the instrument’s weight and comfort less favorably than male participants. Free-text comments suggested that hand size and grip ergonomics influenced these perceptions, underscoring the importance of accommodating anthropometric diversity in device design. A qualitative content analysis categorized the interview responses into key themes. The key findings of the themes are summarized in Table 2 and highlighted with quotes.

**Table 2:** Key findings with quotes from the qualitative content analysis

Theme	Key insights	Quote
Device improvement	Ergonomic limitation: Several participants reported that the device’s grip size and scissor mechanism required excessive force, leading to discomfort during prolonged procedures	“You need to apply too much force, making it harder to use for long surgeries.” (Participant 4)
	Material durability: Concerns were raised about the wear and tear of critical components, particularly in the lower handle	“The lower handle tends to wear out faster than expected.” (Participant 6)
	Navigation screen overlay issues: The device’s integrated screen sometimes obstructed visibility, affecting precision during surgery	“When you open it, the screen overlays the critical area, making it difficult to navigate.” (Participant 5)
	Modular and interchangeable design: Participants suggested a modular approach allowing interchangeable tools instead of replacing the entire device due to minor defects	“Make the tools interchangeable in case of a defect instead of replacing the entire device.” (Participant 3)
	Inclusivity in design (gender bias concerns): Some users felt the device’s design catered predominantly to larger hands, possibly disadvantaging certain users	“Maybe it’s a gender thing, but I found the grip a bit too wide.” (Participant 7)
Device benefits	Quick learning curve and ease of handling: Surgeons found the instrument intuitive, reducing the time needed for training and adaptation	“The experience of learning this instrument was much quicker compared to others.” (Participant 7)
	Enhanced precision and lighter handling: The instrument allowed for greater surgical accuracy, ensuring more controlled movements	“It’s precise when you cut, making fine adjustments easier.” (Participant 6)
	Fast responsiveness: Participants noted immediate feedback and control, improving surgical performance	“Very interesting how quickly it reacts to my movements, almost instant.” (Participant 5)
	Seamless instrument transitions: The ability to switch instruments without losing positioning was highlighted as a major advantage	“I can change instruments without disrupting my position, which saves time.” (Participant 4)
	Greater autonomy for surgeons: Some users reported that the instrument reduced the need for verbal coordination with assistants, streamlining procedures	“No need for communication with assistants for simple instrument adjustments.” (Participant 3)
Areas of application	General surgical applications: Many participants believed the device could be used across all surgical fields, making it a versatile tool	“The device is good for all general surgery procedures.” (Participant 7) “It can be used for everything.” (Participant 5)
	Specific procedures where the device excels: Several respondents provided specific examples where the device proved particularly useful, such as: Upper GI procedures (e.g., gastrectomy, bariatric surgery), hysterectomy or sentinel lymph node dissection	“Good for upper GI (gastrectomy) and bariatrics.” (Participant 7)
	Complexity of procedures: The device was described as effective for both complex and routine surgeries, making it suitable for a wide range of cases	“Good for more complex procedures.” (Participant 7) “It can be used for easier and short operations.” (Participant 4)
Endorsement	High level of endorsement: All participants provided clear and affirmative endorsements of the device	"Absolutely, yes.” (Participant 6)
	Workflow and efficiency improvements: Several respondents emphasized how the device would enhance surgical efficiency and workflow	“Yes, it would make my workflow a lot smoother.” (Participant 3)
	General acceptance and willingness to adopt: The overall sentiment reflected confidence in the device’s potential and a willingness to integrate it into surgical practice	“Yes, I would endorse it.” (Participant 4)
Multi-tool vs. energy device	Potential for multi-tool to replace energy devices: Some participants suggested that multi-tools could reduce dependency on energy devices in certain procedures	“If you have the multitool, maybe you will not often need the energy device.” (Participant 7)
	Energy devices for complex cases: While multi-tools offer versatility, energy devices remain preferred for more advanced or complex procedures	“Energy devices are used for more complex cases.” (Participant 4)
	Multi-tool as an additional or complementary instrument: Many participants did not see multi-tools as a full replacement but rather as an additional tool that enhances flexibility	“The energy devices (LigaSure, etc.) are different, but the multitool can be a good complement.” (Participant 7). “Multitool is on the same level as the energy device but serves a different purpose.” (Participant 6)
Price of multi-tool	€1000 is acceptable for specialized applications: Some participants felt that a €1000 price point could be justified for highly complex or specialized procedures, where the multi-tool provides significant added value	“€1000 is okay for very selected big/complex cases.” (Participant 7); “€1000 is probably fine in some situations.” (Participant 5)
	€1000 is considered too expensive for routine use: Several respondents expressed concerns that €1000 is too costly for widespread use, suggesting that pricing adjustments might be necessary for broader adoption	“€1000 is very expensive. It may be reasonable in some cases, but not generally.” (Participant 6); “€1000 is too much.” (Participants 3 & 4)
Prototype options	Prototype 1 (with wheel)—positive but needs refinements: Participants appreciated the wheel-based control but suggested improvements in its size and flexibility	“I imagine having a screen in addition to the wheel.” (Participant 7); “The size of the wheel needs improvement for better grip.” (Participant 6); “The wheel allows for more flexible rotation in instrument handling.” (Participant 4)
	Prototype 2 (without wheel)—button-based control preference: Some participants preferred button-based controls but suggested adjustments in button positioning to improve usability	“Change the position of the buttons for better access.” (Participant 7); “Prefers the prototype with buttons, not the wheel.” (Participant 3)
Tool requirements for surgery	Preference for pedal-based energy activation: Many surgeons expressed a strong preference for pedals to control energy activation, citing familiarity and habit from existing devices	“Pedals for energy activation because of habit.” (Participant 7); “Pedals are preferred for energy activation.” (Participant 4)
	Essential instruments needed for integration: Participants identified specific instruments that should be included for seamless integration into current surgical practices, including: Bipolar Grasper, Overholt clamps, Scissors	“Instruments needed: Bipolar grasper, Overholt clamps, scissors.” (Participant 6)

Additional references 3 provide detailed reporting of themes with all quotes.

The qualitative content analysis revealed several recurring themes (Additional Reference 3). The most frequently coded category was Device Improvement (48 occurrences, 27%), which mainly concerned ergonomic refinements, haptic feedback, and the positioning of buttons and scissors. For example, one participant remarked: “The scissors are quite weak” (Participant 4), while another noted: “The lower handle feels very light and too exposed to rupture” (Participant 6). The second most frequent theme was Device Benefits (33 occurrences, 18%), with users highlighting rapid and precise tool exchange, autonomy from assistants, and overall workflow facilitation. Illustrative positive comments included: “So I think it could be a revolution in the field of surgery.” (Participant 7), “Yes, it would make my workflow a lot smoother and would be a very big step in the right direction.” (Participant 3), and “I would use it tomorrow.” (Participant 2).

Other themes included suggested areas of application in different surgical fields (18 occurrences, 10%) and comparisons with existing energy devices (15 occurrences, 8%). The complete codebook with all subcodes and explanations is provided in Additional Reference 4. A summary of the distribution of coding across themes is presented in Additional Reference 5, while Additional Reference 6 illustrates the distribution of codes by participant.

## Discussion

This study evaluated the usability, reliability, and safety of a novel surgical prototype across a series of standardized tasks performed by experienced participants. All seven participants completed all tasks successfully, with no safety incidents reported, and the device was generally well-received. Quantitative performance metrics, such as task duration and tool-changing times, suggested consistent operation with low error rates. While occasional software errors required system reboots, these incidents were infrequent and did not compromise procedural completion. Additionally, qualitative feedback highlighted strong enthusiasm for the device’s potential to streamline workflows and enhance surgical efficiency.

Task durations and tool-changing times exhibited relatively low variability, suggesting a minimal learning curve and rapid operator adaptation. Notably, most participants showed stable changing times throughout each task, with only one statistically significant trend observed—participant 6 demonstrated a moderate negative correlation during task 3, possibly indicating increased fluency with the interface. While this isolated finding warrants further exploration, the absence of significant learning trends suggests that the device can be readily used without extensive training.

Tool-change accuracy was high for a pre-clinical prototype, with 4.73% of changes involving incorrect instrument selections. The participants consistently identified and corrected these errors, indicating effective recognition and recovery. Nevertheless, even this proportion underscores the need for further refinement of the user interface and feedback systems to help prevent misselection entirely. Optimizing interface clarity and incorporating stronger haptic or visual confirmation cues may reduce cognitive load during multitasking scenarios. In addition, nine software errors occurred during tool changes, requiring the system to be rebooted in approximately 6% of all changes. While these incidents did not result in procedural failure or user harm in the simulated setting, they represent a substantial limitation. Importantly, a 6% software failure rate cannot be considered acceptable for clinical use, as even rare device malfunctions can disrupt workflow and compromise patient safety. The ultimate goal for future clinical versions of this device must therefore be a near-zero software failure rate, in line with expectations for surgical-grade equipment. It is also important to distinguish between the two types of errors observed: device-related malfunctions (software errors requiring reboot) and user-related mis-selections (incorrect tip choice, immediately recognized and corrected). While their combined incidence was indeed > 10%, they represent different categories of error with different implications for device development. Future iterations of the prototype will thus focus both on improving software stability and on refining the user interface to minimize the risk of misselection. Planned measures include enhancing firmware reliability, implementing redundant error-handling protocols, and streamlining the reboot sequence to avoid dependence on non-sterile assistance. Addressing these issues will be essential to align the device’s performance with its intended goal of reliably improving surgical workflow and efficiency.

Another important finding relates to gender-specific differences in questionnaire responses. Female participants more frequently highlighted issues related to instrument weight and handling comfort, with free-text comments pointing to the role of hand size and grip ergonomics. These observations suggest that gender and anthropometric diversity may affect usability and should be taken into account in the iterative design process. Ensuring that surgical instruments are adaptable across different user populations will be essential for broad adoption.

The statistical modeling revealed no significant association between median tool-changing time and overall task duration. This analysis explored whether the novelty of handling the prototype—reflected in longer instrument-changing times—might have contributed to prolonged task completion. However, both the GAM and GAMM models indicated that tool-changing time was not a meaningful predictor of total task time. These findings suggest that the tasks’ complexity, rather than the inefficiencies introduced by the prototype, primarily determined overall performance. Moreover, the consistently short changing times observed across participants further support the conclusion that the prototype did not impose a temporal burden.

Precision testing of the monopolar application yielded encouraging results. All energy applications were placed accurately, and no safety concerns were reported.

The qualitative analysis added further depth to the evaluation, capturing user perspectives on the prototype’s potential. Surgeons emphasized the device’s potential capacity to streamline surgical procedures and expressed strong enthusiasm for its clinical application. Dominant themes such as “Device Improvement” and “Device Benefits” highlight both a readiness for adoption and a collaborative mindset toward iterative refinement. The strong endorsement—reflected in statements like “I would use it tomorrow”—speaks to the innovation’s perceived value and potential clinical relevance.

Another key theme from the interviews was comparing the prototype to conventional energy devices such as LigaSure© and Harmonic©. Participants were divided in their opinions—some believed the prototype could potentially replace these devices. In contrast, others believed it would serve as a complementary tool, given the well-established benefits of traditional energy systems. This discussion is expected to gain further relevance if the multi-tool proves to be significantly more cost-effective than existing energy devices, potentially shifting the balance in favor of broader adoption.

### Future work

Integrating multifunctional devices, such as the Symphera System, represents a significant advancement in the pursuit of surgical efficiency [20]. Unlike traditional single-function laparoscopic instruments that require frequent exchanges—each taking up to 15–20 s—the Symphera device enables seamless in-body switching between up to eight tool tips in under 3.5 s, potentially reducing tool change time by up to 85% and minimizing workflow disruptions.

As reported in the literature, similar innovations have been associated with measurable improvements in efficiency, cost, and safety. For example, the use of multifunction tools has been shown to reduce total surgery time by up to 17%, depending on the procedure type [11]. Operating room time itself carries significant economic implications, with mean costs estimated at ~ $37 per minute in large-scale analyses of U.S. hospitals [21]. Moreover, prolonged operative times are consistently linked to higher complication rates, with meta-analyses demonstrating a ~ 14% increase in complication risk for every additional 30 min and a doubling of risk once procedures exceed two hours [15]. It is important to emphasize that these figures are literature-based estimates and not derived directly from the present study and that the in this study reviewed device is still an investigational device, not yet approved by the FDA or other regulatory authorities. While our data confirm that intracorporeal tool switching can be performed in < 3.5 s, the downstream clinical and economic benefits remain hypothetical at this stage. Prospective clinical trials and health-economic evaluations will therefore be essential to determine the actual impact of this technology on operative time, complication rates, and hospital costs. Moreover, by consolidating up to 80% of commonly used instruments into one platform, the system addresses ergonomic fatigue and resource overutilization—two often overlooked contributors to surgical burnout and institutional cost inefficiency. These findings are especially pertinent for high-volume, prolonged, or technically complex laparoscopic cases where manual tool switching can significantly impair operative flow and surgeon performance [12, 13]. As such, devices like Symphera may help bridge the gap between conventional laparoscopy and robotic-assisted surgery, offering comparable workflow benefits without high capital investment. They should also be considered part of broader institutional strategies to enhance operative value and patient safety, once cleared by FDA or other regulatory authorities and if clinical trials ultimately demonstrate that they indeed improve surgical performance.

### Limitations

Despite these promising findings, several limitations must be acknowledged.

It is important to note that all results reported here stem from a pre-clinical evaluation conducted in a simulated environment using excised animal tissue using an investigational device, not yet approved by the FDA or other regulatory authorities. Therefore, findings reflect the performance and usability of the prototype under controlled, non-clinical conditions and may not fully capture the complexity of real-world surgical settings.

Lastly, positive bias may influence interview data due to novelty effects or participant enthusiasm.

Future work should focus on expanding the evaluation to a larger, more diverse user group and testing the prototype under more realistic, time-sensitive conditions. Longitudinal studies assessing performance across repeated use will be critical to understanding learning curves and long-term usability. Additionally, technical improvements to enhance system stability and reduce tool misselection will further strengthen the device’s clinical readiness.

## Conclusion

This study demonstrates that the tested Symphera prototype is safe, usable, and well-received by expert users. While further development is warranted, especially regarding software stability and user interface design, these initial pre-clinical results point to an up-and-coming device with the potential to enhance surgical precision and workflow efficiency.

## Supplementary Information

Below is the link to the electronic supplementary material.

Supplementary file1 (DOCX 6740 KB)
