# Supplementary material for: Redefining surgical workflow efficiency: evaluation of a novel laparoscopic multi-tool prototype
Source: Surg Endosc. 2025 Nov 3;40(2):1037–48. doi: 10.1007/s00464-025-12314-y (PMC12880990; doi:10.1007/s00464-025-12314-y)
Supplement: Supplementary file 1 — Supplementary file1 (DOCX 6740 KB) [file 464_2025_12314_MOESM1_ESM.docx]

Additional references 01: Task set up


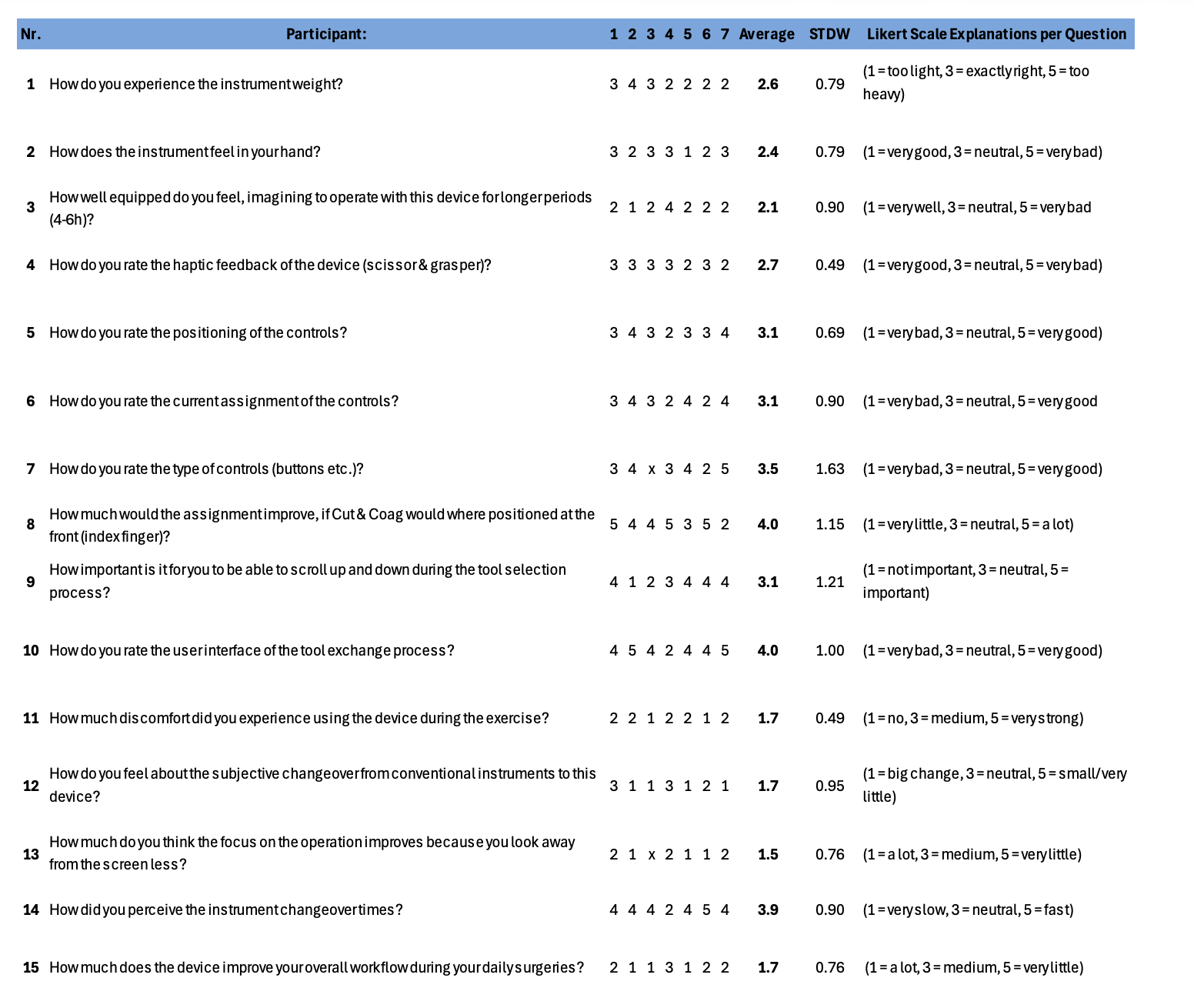


Additional references 02: Complete results of the questionnaire

|  | **Device Improvement** |
| --- | --- |
| **Documents and variables** | **Device Improvement** |
| 7_otter_ai | - Gender bias in design ("so the maybe, maybe the I am female, as in female surgeon. So maybe it was too big. A bit too big for my, for my hand. I had difficulties in scrolling") - Energy activation on pedals not device ("I think that I will always use the pedals") - Button position ("If I don't have the pedal, I will prefer to have the lateral position of for the cut.") - Durability of material ("the lower handle that you have to open and close feels very too light and too. exposed to unexpected rupture. so you can disrupt the handles very easily.") - Missing haptic feedback ("you cannot have a very nice feeling that you are holding an tissue") - Button position ("another button to use, the cut"; "the yellow one on the side. I don't like the position.") - Navigation screen overlay ("So it's also another point but ideally, something that appears widely, I like in the video game, while you are changing your arms.") - Energy activation via pedals ("the fact of pushing on the handle can give you a moment in the deep so this is also preference of the surgeon. Normally, that's also why laparoscopy is the better, because you detatch the hand from the coagulate. so you don't trasfer your motion to the tip of this.") - Too many buttons ("Too much control for two reasons, too much controls in the hand can be press accidentally") - Ergonomy - Scissory quality ("but you need to improve the instruments, because the scissors are quite weak") - Energy activation on the same device ("I don't like to have cautery on the same hand") - Complexity ("closing the same time and choosing can be a little bit difficult, three things to do with too much") - Weight ("perhaps a little bit too light for me.") - Button position ("Well, I think the buttons are not the best place for me") - Interchangable tools in case of defect ("Make it so that the scissors tool can be replaced during operation in case it gets blunt") - Navigation screen overlay ("No extra screen for tool selection, overlay in main operating screen better") - Gender bias in design ("only works for big hands") - Navigation menu ("Number the tools in the tool selection screen so that it is easy to see how many times you have to press to get a certain tool") - Quality of scissors - Energy activation with pedals - Button positioning ("Buttons for cut, coag, and selection are too far away") - Sequence of instruments ("And I will place the bipolar, probably before the scissors, when, when you, when you select the the order") - Deselect instruments ("maybe if you can select that, you can skip some instruments, like five instruments, but you know that you will never use the bipolar or never use the scissors, and that you can deselect them. So then one is, switch is, which. so can make it faster") - Delay in response of handle - Handle should not have to be closed to be able to activate hook - Haptic feedback ("more haptic feedback when you when you cut, especially when you cut, also when you grab things, when you grab things") - Feeling of the buttons ("Maybe I didn't feel very well the Cut button, yellow button") - Ergonomy ("the ergonomic so maybe it will need some little adjustment") - Quality of scissor - Button positioning ("the only limitation I see is basically the button for the coagulation and the cutting") |
| 6_otter_ai |  |
| 5_otter_ai |  |
| 4_check_otter_ai |  |
| Transcript 3 |  |
| 2_with add on_check_otter_ai |  |
| 1_check_otter_ai |  |
|  | **Device Benefits** |
| **Documents and variables** | **Device benefits** |

| 7_otter_ai | - Quick learning of device handling ("the experience was very fast to try the opportunity to try this device.") - Improves autonomy of surgeon ("you can change instrument very fast without the need of ask all the times to the nurses"; "the surgeon can control a little bit more.") - Speed ("because it helps to reduce the time during the operatory room") - Additional screen/interface to select instrument - Precision ("It's precise when you cut") - Light weight ("of course, it's very light. overall It's not heavy, so you can use it for, I think, hours") - Ergnonomy - Overall design - Energy activation on tool/with buttons ("like the buttons. Okay, so you can relax your feet, and you don't have to use also the feet every time, all the in your hands.") - Hook with pistol grip - Change instruments without loosing position ("it's amazing that you can use, like, you can jump from hook to scissor." - Responsive/quick reaction ("So very interesting, the feedback can that you have doesn't bother you so much, and if you are really schematic, soon after making the change. Very responsive motion") - Speed ("the fact that the changing is really can see the improvement of the changes fast is very accurate.") - Changing instruments without loosing position ("What I like for the device that you can change the instrument without taking out") - Grasper functionality - Autonomy ("No need for communication with assistance") - Speed ("The fast instument changes") - Interface to choose instruments ("And I The other thing I found very nice was the interface in front. Because, yeah, then at the end, you understand that the technology, but you don't have any feedback here on the instrument before the instrument gets out") - Energy activation on device, especially for the hook ("I Personally, really like it, especially when you use the hook, because I think I can work better") - Handling ("The thing was very good, even with gloves, like normal setting."; "but I most of the time I didn't have to look at the device to change.") - Balance ("Feels very balanced when you have it in your hand.") - Changing instruments without loosing position ("changing the instrument inside. I mean, I like that.") - Ergonomic ("So I find that it's well, it's well, put in place, it's ergonomic") - Workflow improvement - Safety improvement - Speed ("it can help to speed up the procedure") - Overall design ("can see that the device is well designed") - Changing instruments without loosing position ("act that quickly you can exchange the instrument by staying inside the working field") |
| --- | --- |
| 6_otter_ai |  |
| 5_otter_ai |  |
| 4_check_otter_ai |  |
| Transcript 3 |  |
| 2_with add on_check_otter_ai |  |
| 1_check_otter_ai |  |
|  | **Areas of Application** |
| **Documents and variables** | **Surgeries you can use the device for Specific examples Complexity** |
| 7_otter_ai | The device is good for all general surgery procedures Good for upper GI (gastrectomy) and bariatrics. Also good for Good for more complex procedures. colorectal and emergency surgery. |
| 6_otter_ai | Hysterectomy or sentinel lymph node dissection. |
| 5_otter_ai | It can be used for everything. Abdominal wall surgery, gallbladder, colorectal, gastric, hepatic surgery. |
| 4_check_otter_ai | It can be used for all procedures. It can be used for easier and short operations. |

| Transcript 3 |  | | |
| --- | --- | --- | --- |
| 2_with add on_check_otter_ai | Simple operations like hernia surgery. For cholecystectomy. Upper GI | | |
| 1_check_otter_ai | Colorectal, bariatric, upper GI surgery, Liver surgery. Not for For all advaced cases. gallbladder surgery because 90% of the work is done by a single hook. | | |
|  | **Endorsement** | | |
| **Documents and variables** | **Yes** | | |
| 7_otter_ai | Endorsement ("Yeah!")  Endorsement ("Absolutely, yes.")  Endorsement ("yeah, why not. Its a good device ")  Endorsement ("Yes I would. ")  Endorsement ("Yes, it would make my workflow a lot and would be a very big step in the right direction ")  Endorsement ("Yeah, sure sure sure. ") | Endorsement ("I would use it tomorrow.", "Sure, yeah, I would use it i would use it ", "and yes, yeah, definitely.etely . I would use it") |  |
| 6_otter_ai |  |  |  |
| 5_otter_ai |  |  |  |
| 4_check_otter_ai |  |  |  |
| Transcript 3 |  |  |  |
| 2_with add on_check_otter_ai |  |  |  |
| 1_check_otter_ai |  |  |  |
|  |  | **Multitool vs Energy Device** |  |
| **Documents and variables** | **Competing instruments** | **Multitool can replace** | **Multitool additionally** |
| 7_otter_ai |  | If you have the multitool maybe you will not open an energy device | The energy devices (Ligasure etc) are different concepts. The energy devices are the most important.  You need the energy devices to be faster. |
| 6_otter_ai |  |  | Multitool is on the same level as the energy devices |
| 5_otter_ai |  |  | The advantage of the energy device is that it seals and cuts at the same time |
| 4_check_otter_ai | Energy devices are used for more complex cases |  |  |
| Transcript 3 |  |  |  |
| 2_with add on_check_otter_ai |  | You hesitate to open energy devices for easy cases because the device is very expensive. But you can go faster with an energy device. |  |
| 1_check_otter_ai | Unsure if the multitool can replace an energy device | If the bipolar and the scissors on the multitool is good it can maybe replace an energy device | Multitool will be an additional device to an energy device |
|  |  | **Price of multitool** |  |
| **Documents and variables** | **Specific price recommendation** | **1000€ - ok** | **1000€ - too expensive** |
| 7_otter_ai |  | 1000€ is ok for very selected big/complex cases |  |
| 6_otter_ai |  |  | 1000€ is very expensive. It may be reasonable if you can reuse the instrument. |
| 5_otter_ai |  | 1000€ is probably ok | 1000€ is too much |

| 4_check_otter_ai | 1000€ is too much | |
| --- | --- | --- |
| Transcript 3 |  | |
| 2_with add on_check_otter_ai | 1000€ is ok | |
| 1_check_otter_ai | In Europe the Price should not be higher than 500-600€ 1000€ is too much | |
|  | **Prototype options** | |
| **Documents and variables** | **Prototype 2 - without wheel** | **Prototype 1 - wheel** |
| 7_otter_ai | Change position of buttons | Positive feedback ("I imagine to have a screen and scroll with this. I think it's psychologically more easy") |
| 6_otter_ai |  | Positive. Size of wheel needs improvement |
|  |  | ("It's more seamless, easier to go and rotate a wheel compared to select two different button") |
|  |  | ("yeah, I want, I want this wheel to be at least or bigger or bigger, or with the teeth more pronounced") |
| 5_otter_ai | If an additional screen is required the participant would prefer an instrument with buttons to select the instrument. Not a wheel | Requires a visual help to understand which instrument is selected |
| 4_check_otter_ai |  | Wheel allows more flexibel rotation in instrunment options |
| Transcript 3 | Prefers the prototype with buttons, not the wheel. Buttons to navigate the menu in both directions should be added. |  |
| 2_with add on_check_otter_ai |  | Positive. But wheel needs grip to prohibit slipping and not being able to choose specific instrument. |
|  |  |  |
|  |  | ("But I think sometimes maybe you can miss, I mean, if there are liquids on the field") |
| 1_check_otter_ai | Prototype with buttons is better than with a wheel |  |
|  |  |  |
|  | ("But I think that if you design such a new device, is better that you design it with all the but this solution is better than the wheel, okay, in my opinion, more intuitive") | |
|  | **Tool requirements for surgery** | |
| **Documents and variables** | **What the Surgeon requires** | |
| 7_otter_ai | Pedals for energy activation because of habbits | |
|  | ("Maybe it's for this that you always use the pedals, because in my mind, when I see the hook I have to push with my feet, something. for the bipolar that is the same things in my mind.") | |
|  |  | |
|  | A bipolar is needed | |

6

_otter_ai

Instruments needed: Bipolar grasper, overholt/maryland

5

_otter_ai

4

_check_otter_ai

Pedals are preferred for energy activation

Transcript 3

2

_with add on_check_otter_ai

1

_check_otter_ai

Instrument needed: Grasper, Hook, Bipolar

Additional refrences 3 : Detailed reporting of themes with all quotes that were coded

Code System

| **Code System** | **Explanation** | **Frequency** |
| --- | --- | --- |
| Code System |  | 181 |
| Important quotes | Special quotes from participants with strong exclamations were marked with this code | 3 |
| Price for instrument | This is the heading code for all price related quotes | 0 |
| Specific price recommendation | All quotes that recommended a specific price for the device | 2 |
| 1000€ - ok | Quotes that stated that 1000€ was adequate for such a device | 4 |
| 1000€ - too expensive | Quotes that stated that 1000€ was too much for such a device | 6 |
| Competing instruments | This is the heading code for all quotes that discussed the issue of alternative devices that play a strong role in the operating room. Especially energy devices like Ligasure or Harmonic | 4 |
| Multitool can replace | Code for quotes that state the the multitool can potentially replace these competing instruments | 3 |
| Multitool additionally | Code for quotes that state that the multitool will always be used in addition to the already established energy devices | 8 |
| Surgeries you can use the device for | This is the heading code for quotes discussing what surgeries the multitool can be used for | 5 |
| Specific examples | This code is for quotes naming specific examples of surgeries | 9 |
| Complexity | This code is for quotes that name the complexity level of surgeries the multitool can be used for. | 4 |
| Device endorsement | This is the heading code for quotes that are related to device endorsement – positive or negative | 0 |
| Yes | All quotes that clearly endorse the multitool | 9 |
| Prototype | This is the header for the quotes concerning the additional prototypes that were shown in addition to the functional prototype | 0 |
| Prototype 2 - without wheel | This is the code for the quotes concerning the 2nd additional prototype that has a turning wheel to choose between the instruments | 0 |
| needs improvement | Quotes stating that this design needs improvements | 1 |
| change menu in both directions | Quotes stating that it would be usefull fort he wheel to allow for changes in both directions of the menu | 2 |
| better than prototype 1 | Quotes stating that the prototype with the wheel was better than the prototype with buttons to navigate the menu | 5 |
| Prototype 1 - wheel | Quotes concerning the first additional prototype that was shown with seperate buttons to navigate the tool changing menu | 0 |
| Ability to navigate menu in both directions | Quotes stating that it was important to navigate the tool changing menu in both directions | 2 |
| needs improvement | Quotes stating that this prototype needed improvement | 6 |
| does not improve problems mentioned via device improvement | Quotes stating that this prototype did not represent an improvement compared with the functional prototype that was used for the excercises | 1 |
| neutral | Quotes that did not express positive or negative thoughts about this prototype | 0 |
| positive | Quotes that stated positive remarks about additional prototype 1 | 9 |
| Device Handling | This is the header for all quotes related to device handling – especially glove use | 0 |
| No gloves | Quotes explaining that no gloves were used to handle the prototype | 3 |
| Glove yes | Quotes stating that gloves were used | 0 |
| Amount of pairs 1 | Quotes explaining that one pair of gloves were used | 4 |
| What the Surgeon requires | Header for quotes that relate to the topic what tools a surgeon requires to perform an operation | 0 |
| Instruments needed | Quotes that specifically name instruments that are needed | 6 |
| maryland vs bipolar | Quotes debating if a maryland was required more than a bipolar grasper | 1 |
| Specific sequence of instrument changes | Quotes stating the specfic needed sequence of instrument changes | 1 |
| Seperate energy activation and working device | Quotes that relate to the issue that participants want a seperate energy activation (eg pedal) method compared to direct energy activation on the device itself | 2 |
| Device benefits | Header for all quotes related to positive aspects about the device | 0 |
| Learning how to use the device | Quotes concerning that it was easy to learn to use the device | 1 |
| Autonomy | Quotes stating that the device provided autonomy compared to using regular devices | 2 |
| Precision | Quotes stating that the device is precise | 1 |
| Weight | Quotes stating that the weight of the device was positive and helped with using the device over a prolonged period | 1 |
| Response / Feedback | Quotes stating that the device had a good response time (eg opening and close of the gun grip resulted in immediate movement of the instruments) | 1 |
| Ergonomy | Quotes that stated the ergonomy of the device was good | 2 |
| Workflow improvement | Quotes stating that the device improved the workflow | 1 |
| Safety | Quotes stating that the device improved safety during the operation | 2 |
| Speed | Quotes stating that the device improved the speed of the operation | 3 |
| Overall design | Quotes stating that the overall design of the device was good | 2 |
| Change screen interface | Quotes stating that the intstument change interface was good | 2 |
| Overlay | Quotes stating that the overlay of the chaging interface was good | 1 |
| Energy activation on device | Quotes stating that energy activation on the same deivce (eg no pedals) was good | 2 |
| Handling with gloves | Quotes stating that the handling of the device with gloves was good | 1 |
| Not complex | Quotes stating that the device was not complex | 1 |
| Hook functionality | Quotes commending the functionality of the hook instrument | 3 |
| Balanced in hand | Quotes stating that the instrument was well balanced in hand | 1 |
| Grasper fuctionality | Quotes stating that the functionality of the grasper was good | 2 |
| Change without loosing position | Quotes stating that changing instruments without loosing position (like it would happen with traditional devices) was an improvement | 4 |
| Device Improvement | Header for all quotes related to negative aspects of the device | 0 |
| Make tools changeable | Quotes stating that it would be helpful if tools were interchangable to enable switching tools if there was a malfunction | 1 |
| Change screen overlay needed | Quotes stating that the instument changing menu has to function as an overlay on the main laparoscopic screen to improve handling and minimize gaze deviation | 2 |
| Bugs in software | Quotes stating that there were bugs in the software requiring a reboot of the device | 1 |
| Sex equality - too big | Quotes stating that there was a sex inequality as the device might be too big for female hands | 2 |
| Handle Feeling/Quality | Quotes stating that the feeling of the device was not ideal or that the quality of the materials was too low | 1 |
| Instrument sequence | Quotes stating that the instrument sequence should be changed | 2 |
| Deselect instrument options | Quotes stating that it would be helpful to be able to deselect specific instruments to facilitate changing | 1 |
| Delay in handle reactivity | Quotes stating that there is a dealy in reactivity of instruments when opening and closing device | 1 |
| Hook activation without closing handle | Quotes stating that it would be good to be able to acivate monopolar energy when using the hook without closing the handle of the device | 1 |
| Haptic feedback | Quotes stating that the haptic feedback was not good when handling tissue | 2 |
| Button feeling | Quotes stating that the feeling of the buttons on the device was not good | 1 |
| Menu navigation | Quotes stating that the instrument changing menu needed improvement | 3 |
| Ergonomy | Quotes stating that the ergonomy of the instrument needed improvement | 3 |
| Scissors cutting ability | Quotes stating that the cutting ablility oft he scissors needed to be improved | 3 |
| Energy activation on same device | Quotes stating that energy activation on the same device was not good | 8 |
| Complexity | Requires direct sight to understand where the buttons are | 1 |
| Amount of buttons | Quotes stating that the amount of buttons was too high | 2 |
| Separate fuctions to improve handling | Quotes stating that separate functions were needed to improve handling | 1 |
| Total Weight - Too Light | Quotes stating that the weight of the instrument was too light | 1 |
| Button Positioning | Quotes stating that the button postioning needed to be improved | 11 |

Additional references 4: Complete codebook with subcodes and explanations


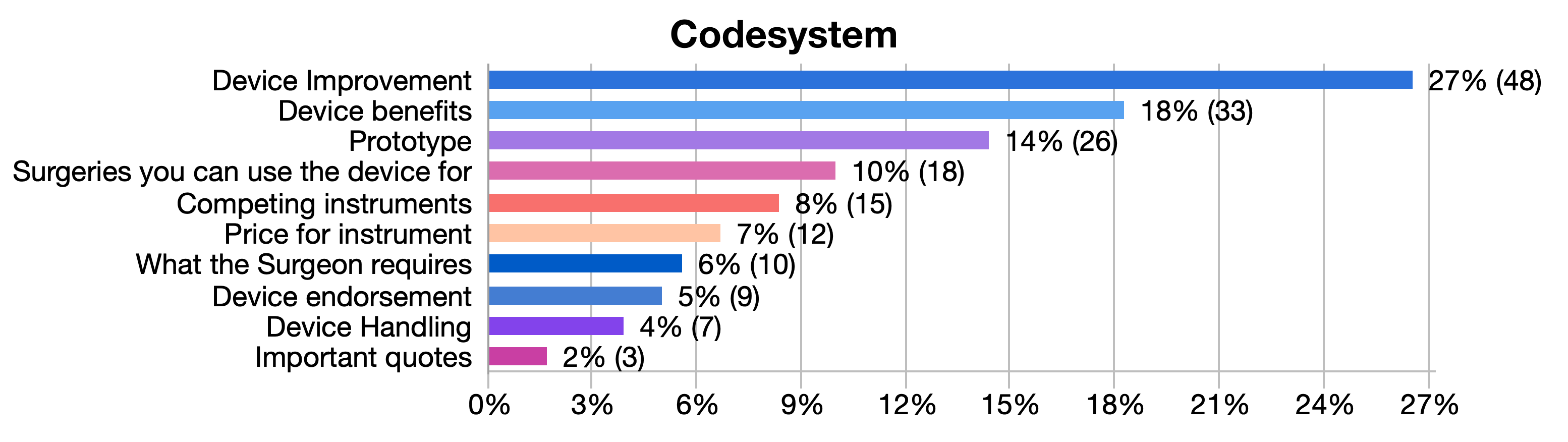


Additional references 5 : Distribution of coding per theme


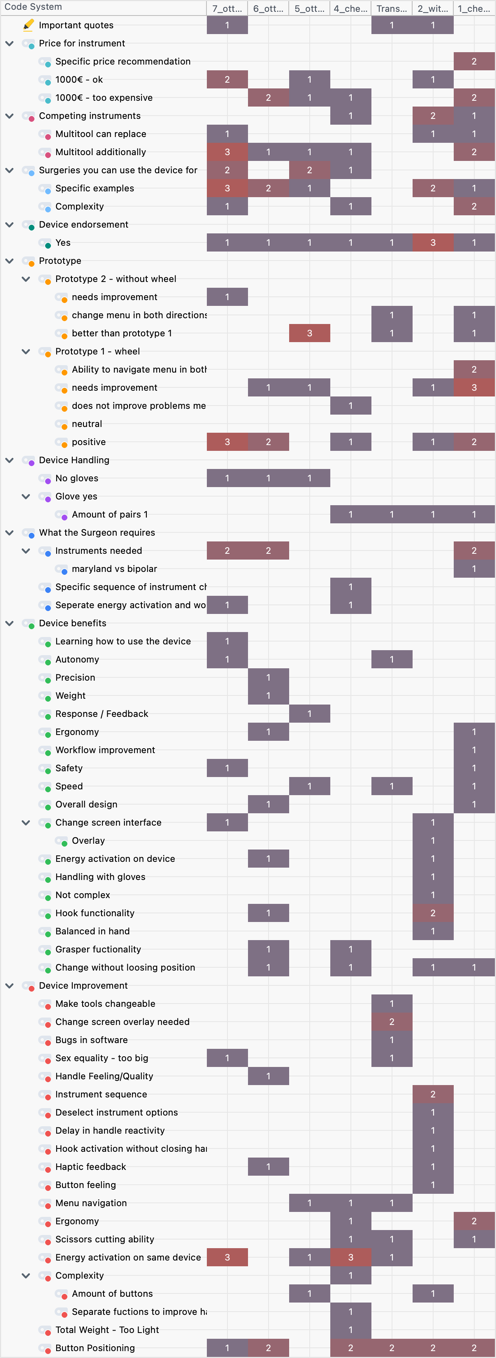


Additional references 6 : Distribution of coding per participant
